# How wearable sensors have been utilised to evaluate frailty in older adults: a systematic review

**DOI:** 10.1186/s12984-021-00909-0

**Published:** 2021-07-08

**Authors:** Grainne Vavasour, Oonagh M. Giggins, Julie Doyle, Daniel Kelly

**Affiliations:** 1 NetwellCASALA, Dundalk Institute of Technology. Co, Louth, A91 K584 Ireland; 2 Ulster University Faculty of Computing Engineering and The Built Environment, Derry(Londonderry), BT48 7JL Northern Ireland

**Keywords:** Wearable sensor, Frailty, Older adults, Physical Activity, Mobility

## Abstract

**Background:**

Globally the population of older adults is increasing. It is estimated that by 2050 the number of adults over the age of 60 will represent over 21% of the world’s population. Frailty is a clinical condition associated with ageing resulting in an increase in adverse outcomes. It is considered the greatest challenge facing an ageing population affecting an estimated 16% of community-dwelling populations worldwide.

**Aim:**

The aim of this systematic review is to explore how wearable sensors have been used to assess frailty in older adults.

**Method:**

Electronic databases Medline, Science Direct, Scopus, and CINAHL were systematically searched March 2020 and November 2020. A search constraint of articles published in English, between January 2010 and November 2020 was applied. Papers included were primary observational studies involving; older adults aged > 60 years, used a wearable sensor to provide quantitative measurements of physical activity (PA) or mobility and a measure of frailty. Studies were excluded if they used non-wearable sensors for outcome measurement or outlined an algorithm or application development exclusively. The methodological quality of the selected studies was assessed using the Appraisal Tool for Cross-sectional Studies (AXIS).

**Results:**

Twenty-nine studies examining the use of wearable sensors to assess and discriminate between stages of frailty in older adults were included. Thirteen different body-worn sensors were used in eight different body-locations. Participants were community-dwelling older adults. Studies were performed in home, laboratory or hospital settings. Postural transitions, number of steps, percentage of time in PA and intensity of PA together were the most frequently measured parameters followed closely by gait speed. All but one study demonstrated an association between PA and level of frailty. All reports of gait speed indicate correlation with frailty.

**Conclusions:**

Wearable sensors have been successfully used to evaluate frailty in older adults. Further research is needed to identify a feasible, user-friendly device and body-location that can be used to identify signs of pre-frailty in community-dwelling older adults. This would facilitate early identification and targeted intervention to reduce the burden of frailty in an ageing population.

## Introduction

Globally the population of older adults is increasing. It is estimated that by 2050 the number of adults over the age of 60 will have almost doubled, representing over 21% of the world’s population [1]. This has huge implications for society not least because of the increase in physical decline and chronic illness associated with ageing.

Frailty is a clinical condition associated with ageing, characterised by multi-system decline resulting in an increase in adverse outcomes such as falls, hospitalisation, institutionalisation and mortality [2]. Fried’s Frailty Phenotype (FFP) [2], the most commonly used frailty assessment tool [3] defines frailty as the presence of three or more of the five identified phenotypes; sarcopaenia, weakness as demonstrated by reduced grip-strength and slow gait-speed, fatigue and reduced level of activity [2]. It is considered the greatest challenge facing an ageing population [4, 5] affecting an estimated 16% of community-dwelling populations worldwide [6] and 21.5% of over 65’s in Ireland [5]. Frailty is associated with, but is not an inevitable part of ageing and it is thought to be transitional. Research suggests that with intervention people can transition between stages of frailty, from pre-frail (PF) to robust or non-frail (NF) and albeit to a lesser extent, from frail (F) to robust [7, 8]. Robust or NF is defined as the absence of phenotypes while PF, considered the prodromal stage of frailty is defined as the presence of one or two phenotypes [2].

The association between physical inactivity and frailty is well documented [9–13]. Physical activity (PA) and physical fitness are inversely related to chronic disease and all-cause mortality, including frailty [14]. As a result, the World Health Organisation has developed guidelines and an action plan to promote PA, healthy ageing and reduce functional decline, with the view to reducing the burden of sequelae of inactivity on both the individual and the health system [15]. More recent guidelines include advice on reducing sedentary time [16]. It is thought however, that only one in four adults over the age of 18 meet guidelines for minimum activity levels [15]. Results for older adults (> 65 years of age) meeting the recommendations varies from zero [11] to between 15% [17] and 87% [18].

Traditionally, measurement of mobility and PA has relied on the use of self-reported questionnaires, surveys or diaries, or direct observation of physical performance tests, each with inherent difficulties and limitations. While these methods can be cost-effective and simple to administer they carry a risk of bias from recall, desire to perform better and participant reactivity, a well-recognised phenomenon of behaviour change due to the awareness of being observed [19].

Recent advances in technology provide the opportunity for objective measurement of mobility and PA through the use of wearable sensors. This allows for unbiased examination of PA patterns and behaviours which can inform guidelines and promote more widespread participation [11, 20, 21]. Wearable sensors are devices that incorporate various technologies capable of physiological, biomechanical and motion sensing. They can be incorporated into shoes and clothing, worn as pendants, attached to the wrist, ankle or trunk, or carried in a pocket. Wireless inertial units are the most commonly used sensors in wearable systems [22]. In the form of accelerometers, gyroscopes, pedometers or heart-rate monitors, wearable sensors have the capacity to measure activity frequency, duration and intensity. Accelerometers measure linear acceleration in real time and can detect movement in up to 3 planes, i.e. vertical, antero-posterior and medio-lateral. Pedometers measure the number of steps taken and correlate well with uni-axial accelerometers [23]. Gyroscopes measure changes in orientation such as rotational or angular velocity, acceleration or displacement. Heart rate monitors are one type of sensor among others capable of capturing indications of physical activities that do not require trunk displacement and can be used to indicate energy expenditure and PA behaviours e.g. sedentary time [24].

Considering the increasing population of older adults, ninety-five percent of who are community-dwelling [25], identifying a way for individuals to independently and objectively monitor their risk of developing frailty is vital. Earlier reviews have reported on the use of wearable sensors in relation to gait analysis [26], falls risk [27], rehabilitation [28] and levels of PA in hospitalised frail elderly [29] and community-dwelling older adults [21]. The aim of this systematic review is to examine the literature to explore how wearable sensors have been used to identify frailty and pre-frailty in older adults and compare with a traditional frailty classification tool. Specifically it aims to discern which parameters of mobility and PA obtained from wearable sensors have been best used to quantify frailty in older adults, the type of body-worn sensors used to provide these parameters, the sensor-placement used and how the parameters of mobility and PA are associated with the discrimination of frailty stages.

## Methods

### Search strategy

This systematic review was conducted in accordance with the Preferred Reporting Items for Systematic Reviews and Meta-Analyses (PRISMA) [30] and is registered with the International prospective register of systematic reviews (PROSPERO) (registration number CRD42020163082). Using the PICO framework (Population, Intervention, Comparator and Outcome) to develop search terms, one investigator searched the electronic databases MEDLINE, Science Direct, Scopus, and CINAHL as per previous reviews [7, 21, 31]. The search was carried out in March 2020 and updated November 24th, 2020 to ensure all recently published articles meeting the criteria were included. The search strategy was developed in consultation with a librarian. The complete search strategy used in MEDLINE and adapted to the other electronic sources is shown in Appendix 1. Reference lists of eligible papers were manually searched for additional studies.

### Study selection

Papers were selected if they were available in English and met the following criteria: Primary observational studies, performed in a laboratory, clinical or free-living (home/community) environment; Recruited older adults > 60 years of age; Involved the use of any consumer, research or medical-grade wearable sensor to provide quantitative measurements of mobility and/or PA, and included a standardised frailty classification tool.

Studies were excluded if they used non-wearable sensors (e.g. ambient sensor) for outcome measurement, or outlined mobility/PA algorithm or application development exclusively.

Titles and abstracts were screened by one investigator. Full texts of studies identified by this review were screened for eligibility by three investigators independently. Consensus was reached through discussion.

### Data extraction

Data extracted from each study included first author, year of publication, number of participants and age profile, study setting, wearable sensor used; make, model and manufacturer, study objectives and methods, parameters of PA/ Mobility measured, frailty measure, reported findings and their statistical analysis. The methodological quality of the selected studies was assessed using the Appraisal Tool for Cross-sectional Studies (AXIS) [32].

### Analysis

Due to the heterogeneity of the study methodology, methods of analysis and outcomes reported, a meta-analyses was not possible for this review.

## Results

### Literature search

**Fig. 1:**
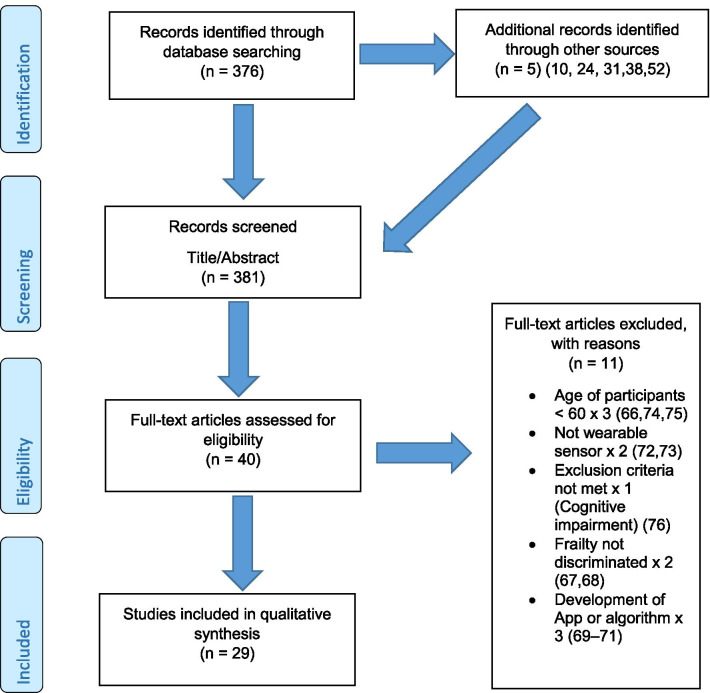
PRISMA 2009 flow diagram

The initial search identified 376 papers published since 2010. Following screening of titles and abstracts and removal of duplicates, 35 articles were deemed appropriate for full text screening. Five further articles were identified from manual search of references of eligible studies. One paper [33] was published after the updated search but was included when discovered incidentally. Of the 40 articles reviewed, 11 were excluded (See Appendix 2). The remaining 29 were included in the review (Table 1). Figure 1 outlines the selection process.

**Table 1:**
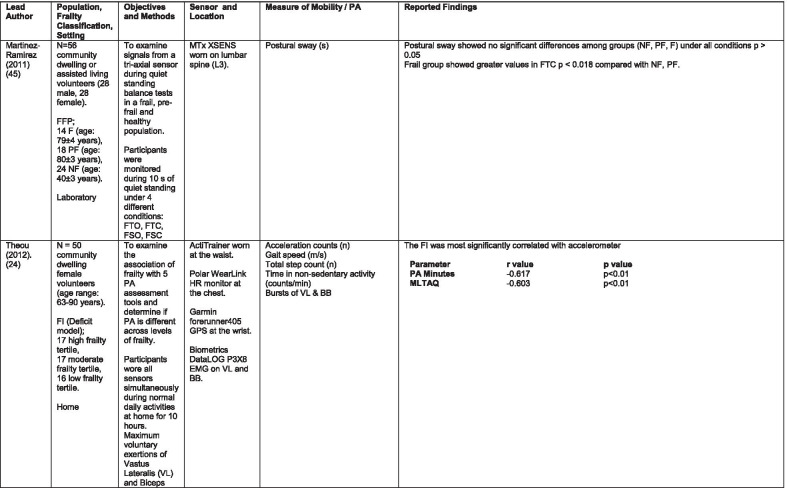

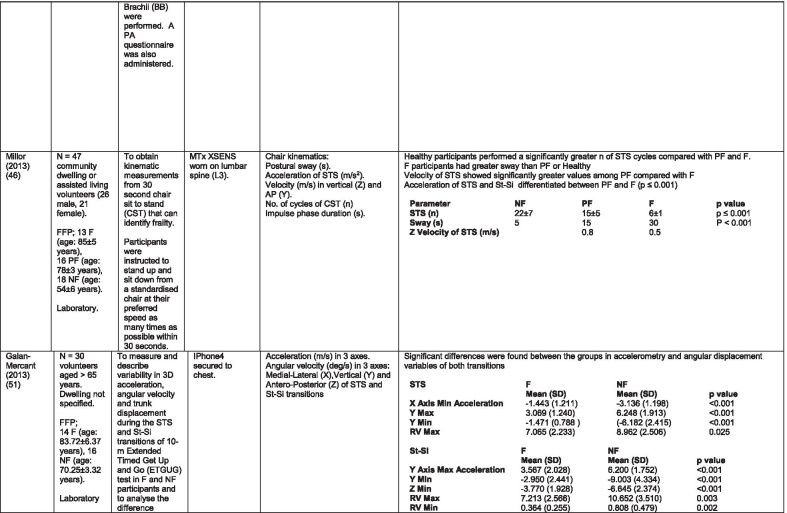

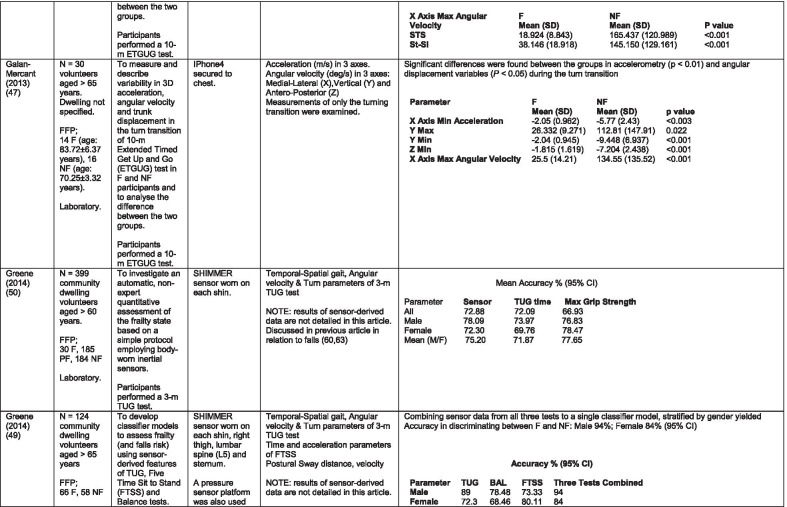

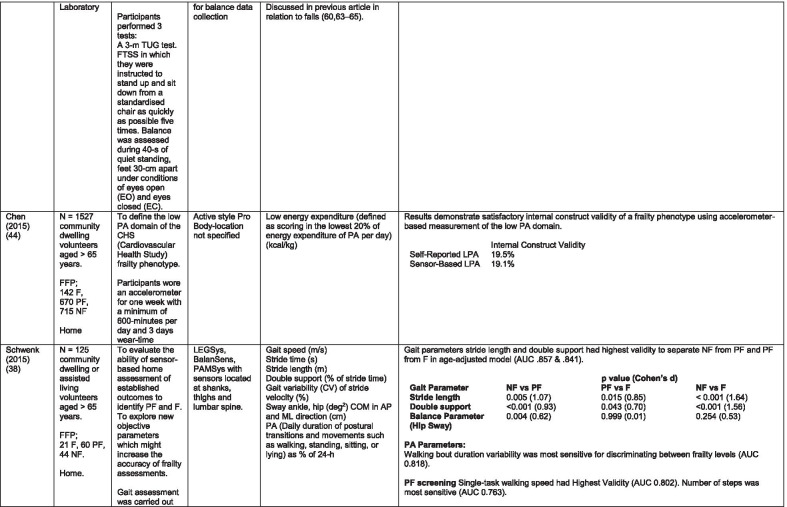

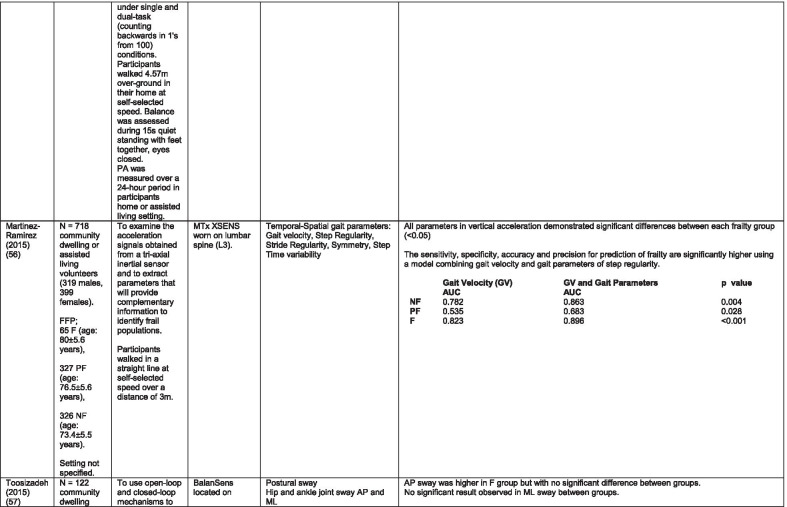

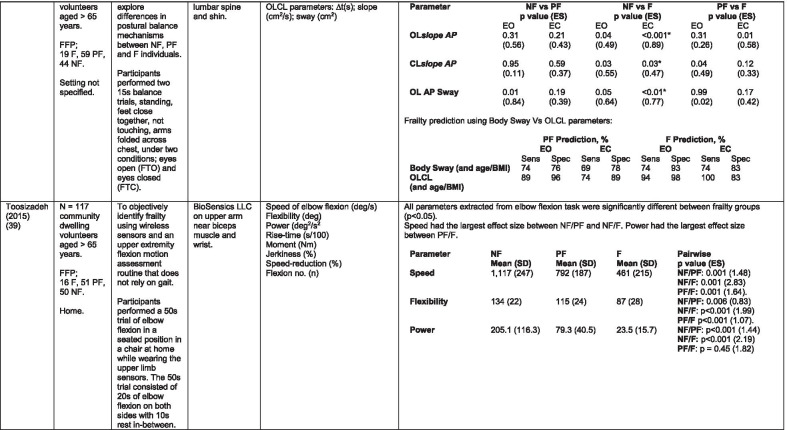

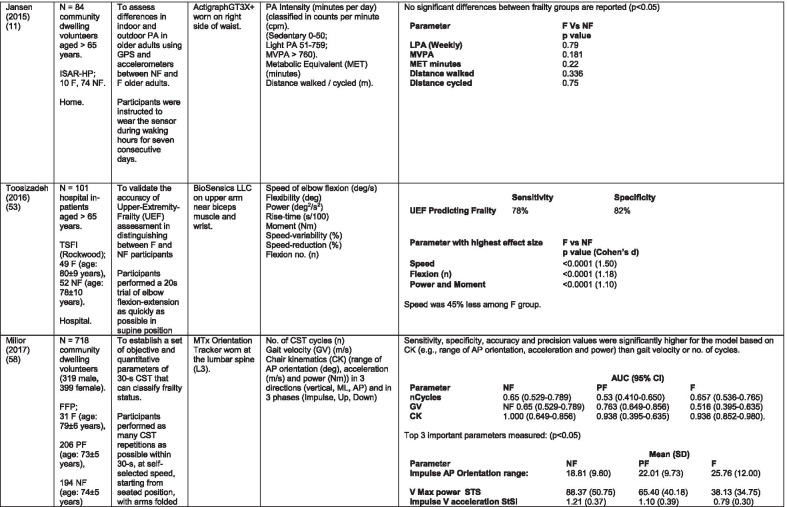

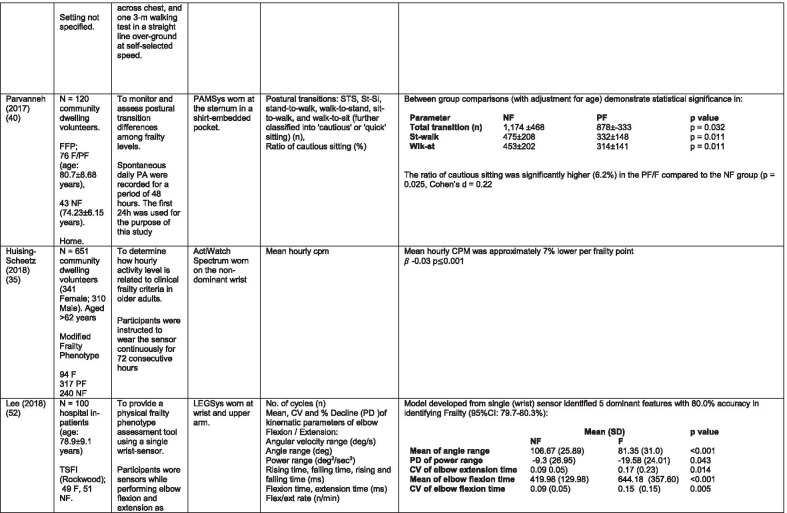

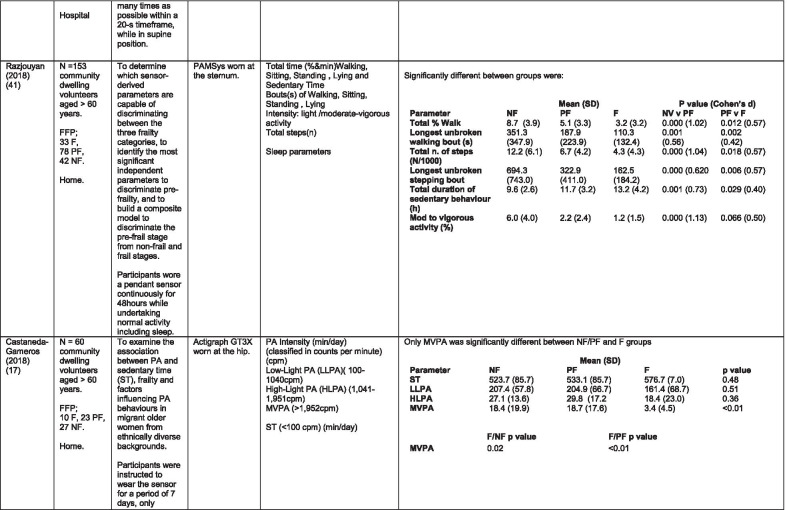

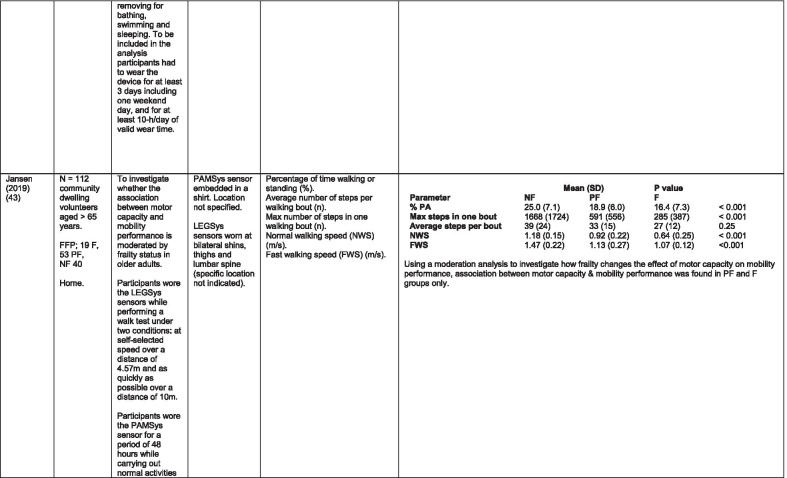

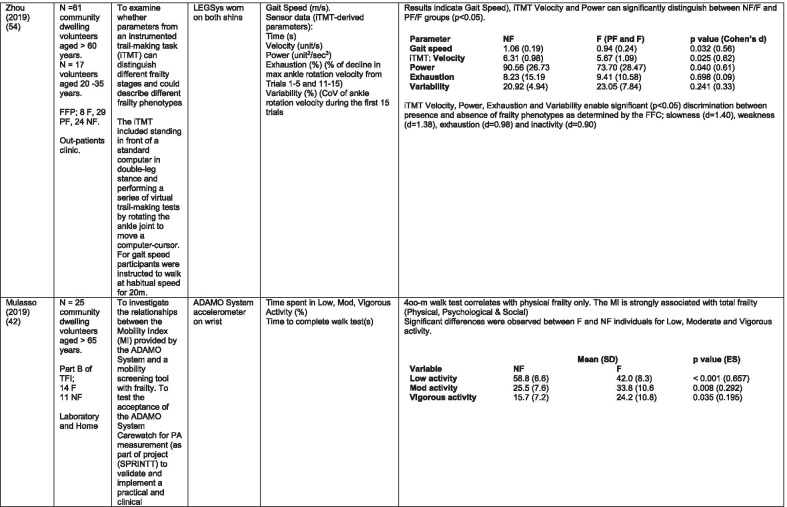

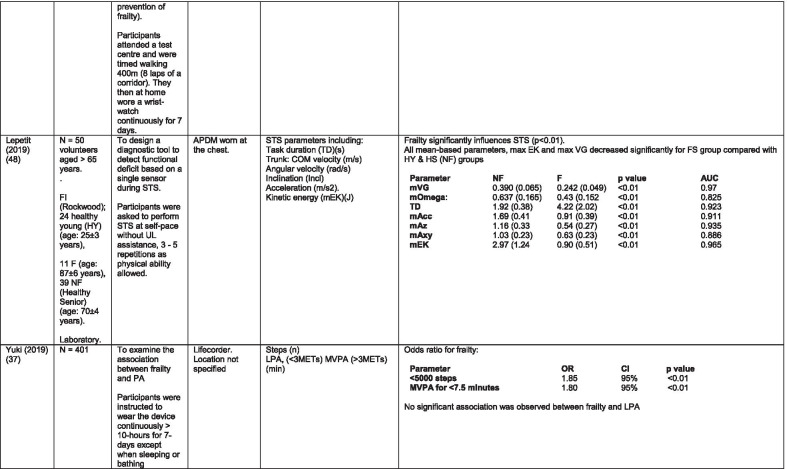

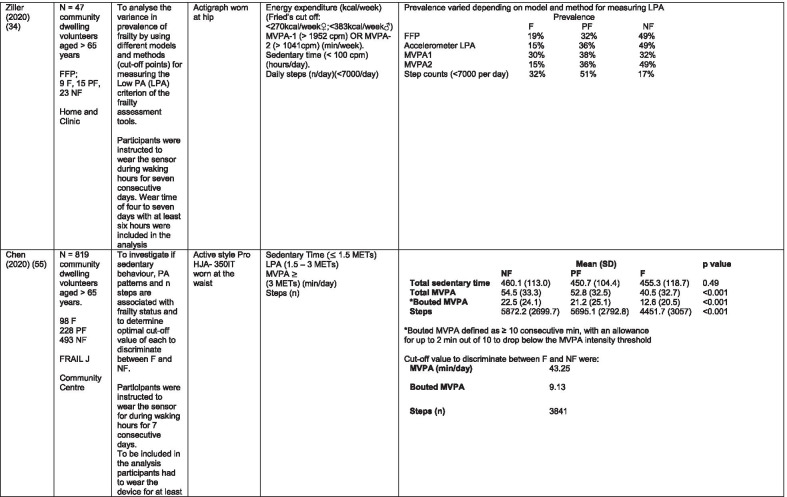

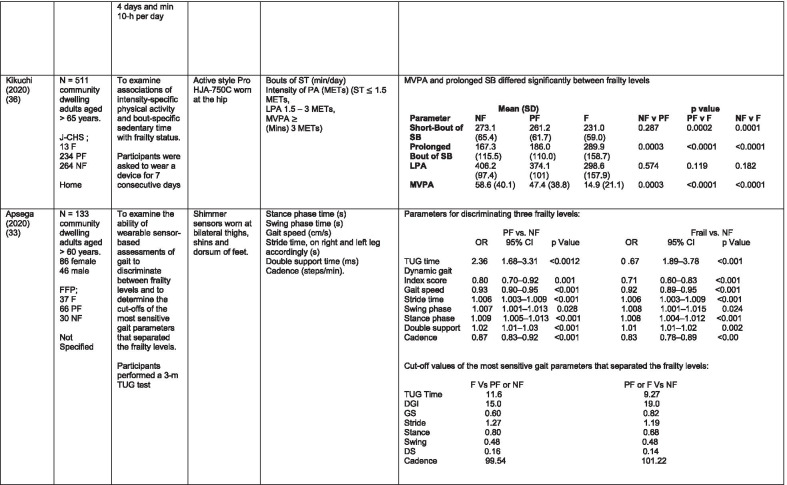
Data extraction

*N/n* Number, *FFP* Fried’s Frailty Phenotype, *F* Frail, *PF* Pre-Frail, *NF* Non-Frail, *s* seconds, *FTO* Feet Together Eyes Open, *FTC* Feet Together Eyes Closed, *FSO* Feet Semi-tandem Eyes Open, *FSC* Feet Together Eyes Closed, *L3* Lumbar Vertebrae n 3, *PA* Physical Activity, *GPS* Global Positioning System, *EMG* Electromyography, *m/s* metre per second, *VL* Vastus Lateralis, *BB* Biceps Brachii, *FI* Frailty Index, *r* Correlation coefficient, *CST* Chair Stand, *cpm* counts per minute, m/s^2^ metre per second squared, *STS* Sit To Stand, *St-Si* Stand to Sit, *3D* 3-Dimensional, *ETGUG* Extended Timed Get Up and Go, *TUG* Timed Up and Go, *MGS* Maximum Grip Strength, *FTSS* Five Times Sit to Stand, *CI* Confidence Interval, *CHS* Cardiovascular Health Study, *kcal/kg* calorie per kilogram, *CV / CoV* Coefficient of Variation, *COM* Centre of Mass, *AP* Antero-Posterior, *ML* Medial–lateral; *h* hour, *AUC* Area Under Curve, *RMS* Root Mean Square, *OLCL* Open Loop Closed Loop; *∆t* Change in time, *MVPA* Moderate to Vigorous PA; *MET* Metabolic Equivalent, *ISAR-HP* Identification of Seniors At Risk-Hospitalised Patients Questionnaire; *TFI* Tilburg Frailty Index, *TSFI* trauma-Specific Frailty Index, *UEF* Upper-Extremity Frailty Assessment; *GV* Gait Velocity, *CK* Chair Kinematics; *SD* Standard Deviation, *ST* Sedentary Time, *LLPA* Low-Light PA, *HLPA* High-Light PA, *NWS* Normal Walking Speed, *FWS* Fast Walking Speed, *iTMT* instrumented Trail-Making-Task, *mVG* Mean value of the norm of the torso COM velocity; mOmega, mean value of the norm of the trunk angular velocity, *TD* Task Duration, *mAcc* mean Acceleration, *mAz* Acceleration in vertical axis; *mAxy* mean acceleration in horizontal plane, *mEK* mean kinetic energy, *Frail-J J-CHS* Frailty Indices adapted for Japanese older adults, *DGI* Dynamic Gait Index, *DS* Double Support

### Study characteristics

All studies included in the review were either validation (< 25%) or observational cross-section design. One study [17] was a mixed methods design but only the objective quantitative results were included in the report. The studies were carried out in varying settings; home: n = 14 [11, 17, 24, 34–44], laboratory: n = 8 [42, 45–51], hospital: in-patient n = 2 [52, 53], out-patient n = 2 [34, 54], community centre n = 1 [55] and not specified: n = 4 [33, 56–58]. Participant numbers ranged from n = 30 to n = 718. Criteria of frailty classification included Fried’s Frailty Phenotype (n = 19) [17, 33, 34, 38–41, 43–47, 49–51, 54, 56–58], modified Frailty Phenotype (n = 3) [35, 36, 55], Rockwood’s Frailty Index (n = 2) [24, 48] Trauma-Specific FI (n = 2) [52, 53], Identification Seniors At Risk-Hospitalized Patients’ questionnaire (ISAR-HP) (n = 1) [11] and Tilburg Frailty Indicator (n = 1) [42].

Of the studies included, 13 different body-worn sensors were used in eight different body-locations. Details of sensors are provided in Table 2. One study used an iPhone as a body-worn sensor by affixing to the chest and was thus included in the study, data from which is presented in two separate articles [47, 51]. Sensor placement included the lumbar spine (LSp) (n = 8), chest (n = 7), shin/ankle (n = 7), wrist and upper-limb combination (n = 3), wrist (n = 2), waist (n = 3), hip (n = 3), thigh (n = 3), foot (n = 1) and not specified (n = 3). Nineteen studies used just one body location [11, 17, 34–37, 40–42, 45–48, 50, 51, 54–56, 58], three studies, measuring elbow kinetics specifically, used a combination of above elbow and wrist [39, 52, 53] while six others used multiple body-locations of LSp and shin [57], and chest, LSp, thigh, shin and foot [24, 33, 38, 43, 49].

**Table 2:** Sensor details

Author (Reference n.)	Sensor type,Location and properties where provided	Acquisition, processing and analysis
Martinez-Ramirez [45]	MTx XSENS,Xsens Technologies B.V. Enschede, Netherlands Tri-axial accelerometer, gyroscope & magnetometer worn at L 3 combines nine individual MEMS sensors to provide drift-free 3D orientation as well as kinematic data: 3D acceleration, 3D (rate gyro) and 3D magnetometers	A wavelet-based algorithm using Fourier Technique, Wavelet Decomposition, Principal Component Analysis
Theou [24]	ActiTrainer Uni-axial accelerometer worn on waist Records data in 1-min epochs Polar WearLink HR monitor worn on chest, Garmin forerunner405 GPS worn on wrist Biometrics DataLOG P3X8 EMG worn on Vastus Lateralis and Biceps Brachii	Data downloaded or wirelessly transmitted to Custom Software EMG sampling frequency 1000 Hz
Millor [46]	MTx XSENSXsens Technologies B.V. Enschede, Netherlands Tri-axial accelerometer, gyroscope & magnetometer worn at L3	Sampling frequency 100 Hz, Automated raw data analysis using Matlab (Mathworks Inc., Natick, MA., USA)
Galan-Mercant [47, 51]	iPhone4 secured to chest Tri-axial accelerometer, gyroscope & magnetometer Apple uses a LIS302DL accelerometer in iPhone4	Sampling frequency 32 Hz. Data obtained through the use of an Application xSensor Pro, Crossbow Technology Inc., available from Apple *AppStore*
Greene [50]	SHIMMER, Dublin, Ireland Tri-axial accelerometer & gyroscope worn on each shin Sensor axes aligned with the vertical, medio-lateral and anterior–posterior axes of the body,	Sampling frequency 102.4 Hz, Low-pass filtered with zero-phase 2nd order Butterworth filter, 20 Hz corner frequency. Raw data analysis using Matlab (Mathworks Inc., Natick, MA., USA)
Greene [49]	SHIMMER, Dublin, Ireland Tri-axial accelerometer & gyroscope worn on each shin, lateral aspect of right thigh, Sternum above L5	Inertial sensor Sampling frequency 102.4 Hz, 2nd order Butterworth filter. Pressure sensor 40 Hz. Raw data analysis using Matlab (Mathworks Inc., Natick, MA., USA)
Chen [44]	Active Style Pro, HJA350-IT, Omron Healthcare, Co. Ltd, Kyoto, Japan) Tri-axial accelerometer. Location not specified	Details not provided
Schwenk [38]	LEGSys™, BalanSens™, PAMSys™ Locomotion Evaluation and Gait System, (BioSensics, Cambridge, MA) Tri-axial accelerometer, gyroscope, magnetometer sensors worn on shanks, thighs, and L	Sampling frequency 100 Hz Custom software LEGSys™, BalanSens™,
Martinez-Ramirez [56]	MTx XSENS,Xsens Technologies B.V. Enschede, Netherlands Tri-axial accelerometer, gyroscope & magnetometer worn at L3	Gait features were detected using automatic peak detection and identified using wavelet decomposition (Coif5 level 3)
Toosizadeh [57]	BalanSens ™ BioSensics (LLC, Brookline, Mass., USA) Triaxial accelerometer, gyroscope, magnetometer worn at shank and trunk	Sampling frequency 100 Hz Real time quaternions were converted to Eular angles
Toosizadeh [39]	BioSensics LLC Tri-axial gyroscope worn on Upper Arm near Biceps muscle and wrist	Sampling frequency 100 Hz Further details of sensor-data extraction not provided
Jansen [11]	ActiGraph GT3X + (ActiGraph, Pensacola, Florida) and BT-Q1000XT (QStarz International Co) Tri-axial accelerometer and GPS receiver worn on waist	ActiLife v5.8.3 Firmware v2.2.0, was used to process accelerometer data
Toosizadeh [53]	BioSensics LLC Tri-axial gyroscope worn on Upper Arm near Biceps muscle and wrist	Sampling frequency 100 Hz Further details of sensor-data extraction not provided
Millor [58]	MTx Orientation Tracker (WSENS, Xsens Technologies B.V., Enschede, Netherlands) Tri-axial accelerometer, gyroscope & magnetometer worn at LSp3	Sampling frequency 100 Hz. Nine individual MEMS sensors provided kinematic data. Drift-free orientation data was also provided using Kalman filters. Automated data analysis using Matlab (Mathworks Inc., Natick, MA., USA)
Parvanneh [40]	PAMSys TM (BioSensics LLC, Watertown, MA, USA), Tri-axial accelerometer worn at Sternum	Sampling frequency 50 Hz. Custom software / algorithm (PAMWare, BioSensics Cambridge, MA, USA)
Huisingh-Scheetz [35]	ActiWatch Spectrum Tri-axial piezo-electric accelerometer worn on wrist	Sampling frequency 32 Hz. Data processed using Actiware® software
Lee [52]	LEGSys™(Biosensics LLC, Watertown, MA) Tri-axial gyroscope worn on wrist and Upper arm	Sampling frequency 100 Hz, Automated raw data analysis using Matlab (Mathworks Inc., Natick, MA., USA). An algorithm was developed using zero crossing technique, with no filtering, to automate phenotype extraction
Razjouyan [41]	PAMSys™ (BioSensics LLC, Watertown, MA, USA) Tri-axial accelerometer worn at sternum	Sampling frequency 50 Hz. The raw data were processed with a band-pass filter at cut-off frequencies of 0.1953 Hz and 12.5 Hz
Castaneda-Gameros [17]	Actigraph GT3X accelerometer (Actigraph, Pensacola, FL) worn on Hip. Programmed to record activity in 60-s epochs	Data were cleaned and scored using ActiLife software V6.2
Jansen [43]	LEGSys™ (BioSensics, Cambridge, Mass., USA) Tri-axial accelerometer, gyroscope, magnetometer worn on shanks, thighs, and L	Algorithm based on accelerometer data with low-pass filtering (as described in author’s earlier publication)
Zhou [54]	LEGSysTM (BioSensics, MA, USA) Tri-axial accelerometer, gyroscope, magnetometer worn on both shins	Quaternion components of ankle rotation were converted to Eular angles. Sampling frequency 100 Hz
Mulasso [42]	ADAMO System (Caretek S.r.l., Turin, Italy) Tri-axial accelerometer worn on wrist	Embedded step-count algorithm. Sampling frequency 50 Hz
Lepetit [48]	APDM (Opal, Portland, USA) Tri-axial accelerometer, gyroscope, magnetometer worn on chest	Fusion algorithm. Sampling frequency 128 Hz
Yuki [37]	Lifecorder (Suzuken, Aichi, Japan) Uniaxial accelerometer. Body-location not specified	Data recorded in 4-s epochs. No further information available
Ziller [34]	ActiGraph wGT3x-BT Tri-axial accelerometer worn at hip	Sampling frequency 100 Hz, 10-s epochs. Data processing using ActiLife Software 6, ActiGraph, LLC
Chen [55]	Active style Pro HJA- 350IT, Omron Healthcare, Kyoto, Japan Triaxial accelerometer worn at the waist	Data recorded in 60-s epochs. No further detail available
Kikuchi [36]	Active style Pro HJA-750C; Omron Healthcare, Kyoto, Japan Triaxial accelerometer worn at the hip	Data recorded in 60-s epochs. Analysis using application developed by Omron Healthcare Co., Ltd to read METs data from accelerometer
Apsega [33]	SHIMMER, Dublin, Ireland Tri-axial accelerometer & gyroscope worn on each thigh, shin and dorsum of foot	Sampling frequency 256 Hz. Butterworth second order low pass filter with an 8 Hz cut-off and an additional least square method 25th order filter with a 10 Hz cut-off for composite foot acceleration data. A gait event detection algorithm was developed

Seven different measures of mobility and PA were reported. Mobility measures included temporal-spatial gait parameters of speed, total steps, double support, stride length, time and variability [24, 33, 38, 47, 49, 50, 54, 56], postural transitions: acceleration counts of sit to stand (STS), stand to walk, stand to sit [24, 40, 41, 46, 48, 49, 58], trunk angular velocity [47, 50], upper limb kinematics [39, 52, 53], intensity of PA and percentage of time in walking, standing, sitting and lying [11, 17, 24, 35–38, 40–43, 55]. Two studies examined PA intensity with the aim to objectively define and compare with the low PA criterion of a frailty classification tool [34, 44]. Balance parameters included sway of ankle, hip and centre of mass [30, 36, 41, 24] and chair-stand kinematics including number of STS cycles, acceleration and trunk displacement [46, 48, 49, 58].

### Participant characteristics

Participants ranging in age 63–90 years were recruited from community, assisted-living or hospital environments. Four studies [45, 46, 48, 54] included a healthy young cohort (age range 18–54 years) for comparison. For those studies that reported sex there was an overall predominance of females.

### Quality assessment

With the exception of one study that scored 12, the methodological quality of studies demonstrated a minimum result of 70% (14 out of a possible 20, range 14–20) using the AXIS tool (Appendix 3). Quality appraisal of all 29 studies is presented in Table 3. The tool used does not apply a numerical score or rating because of the author’s assertion of the non-linear weighting of each aspect of the assessment and each Sect. [59]. No study was excluded based on methodological score.

**Table 3:** AXIS methodological quality assessment

Study	Q1	2	3	4	5	6	7	8	9	10	11	12	13*	14	15	16	17	18	19*	20	Total
Martinez-Ramirez [45]	1	1	0	1	1	0	0	1	1	1	1	1	0	0	1	1	1	1	1	1	15
Theou [24]	1	1	1	1	1	0	0	1	1	1	1	1	0	0	1	1	1	1	1	1	16
Millor [46]	1	1	0	1	1	0	0	1	1	1	1	1	0	0	1	1	1	0	1	1	14
Galan-Mercant [51]	1	1	0	1	1	0	0	1	1	1	1	1	0	0	1	1	1	0	1	1	14
Galan-Mercant [47]	1	1	0	1	1	0	0	1	1	1	1	1	0	0	1	1	1	1	0	1	14
Greene [50]	1	1	1	1	1	0	0	1	1	1	1	0	0	0	1	1	1	1	0	1	14
Greene [49]	1	1	0	1	1	0	0	1	1	1	1	0	0	0	0	1	1	1	0	1	12
Chen [44]	1	1	1	1	1	1	1	1	1	1	1	0	1	1	1	0	1	1	1	1	18
Toosizadeh [57]	1	1	1	1	1	0	0	1	1	1	1	1	0	0	1	1	1	1	1	1	16
Toosizadeh [39]	1	1	1	1	1	0	0	1	1	1	1	1	0	0	1	1	1	1	1	1	16
Schwenk [38]	1	1	0	1	1	0	0	1	1	1	1	1	0	0	1	1	1	1	1	1	15
Martinez-Ramirez [56]	1	1	0	1	1	0	0	1	1	1	1	1	0	0	1	1	1	1	1	1	15
Jansen [11]	1	1	1	1	1	1	1	1	1	1	1	1	1	1	1	1	1	1	1	1	20
Toosizadeh [45]	1	1	0	1	1	0	0	1	1	1	1	1	0	0	1	1	1	1	1	1	15
Parvanneh [40]	1	1	1	1	1	0	0	1	1	1	1	1	0	0	1	1	1	1	0	1	15
Millor [58]	1	1	0	1	1	0	0	1	1	1	1	1	0	0	1	1	1	1	0	1	14
Huisingh-Scheetz, [35]	1	1	1	1	1	1	1	1	1	1	1	1	1	1	1	1	1	1	1	1	20
Lee [52]	1	1	0	1	1	0	0	1	1	1	1	1	0	0	1	1	1	1	0	1	14
Castaneda-Gameros [17]	1	1	1	1	1	0	0	1	1	1	1	1	0	0	1	1	1	1	1	1	16
Razjouyan [41]	1`	1	0	1	1	0	0	1	1	1	1	1	0	0	1	1	1	1	0	1	14
Mulasso [42]	1	1	0	1	0	0	0	1	1	1	1	1	0*	1	1	1	1	1	0	1	14
Zhou [54]	1	1	0	1	1	0	0	1	1	1	1	1	0	0	1	1	1	1	0	1	14
Lepetit [48]	1	1	0	1	1	0	0	1	1	1	1	1	0	0	1	1	1	1	1	1	15
Jansen [43]	1	1	0	1	0	0	0	1	1	1	1	1	0	0	1	1	1	1	1	1	14
Yuki [37]	1	1	1	1	1	0	0	1	1	1	1	1	0	0	1	1	1	1	1	1	16
Ziller [34]	1	1	1	1	0	1	1	1	1	1	1	1	1	1	1	1	1	1	1	1	19
Chen [55]	1	1	1	1	1	1	1	1	1	1	1	1	1	1	1	1	1	1	1	1	20
Kikuchi, [36]	1	1	1	1	1	1	0	1	1	1	1	1	1	0	1	1	1	1	1	1	18
Apsega (33)	1	1	1	1	1	0	0	1	1	1	1	1	0	0	1	1	1	1	1	1	16

AXIS Methodological Quality Assessment (Yes = 1, No = 0, Not known = 0)

*Q 13 “Does the response rate raises concerns about non-response bias?” *Q19 “Were there any funding sources or conflicts of interest that may affect the authors’ interpretation of the results? ‘No’ is a positive response, therefore ‘No’ counts as ‘1’

## Discussion

This systematic review was undertaken to examine which parameters of mobility and PA obtained from a wearable sensor have been used to assess and quantify frailty, which type of body-worn sensors and specific body-locations have been used and how different parameters are associated with discrimination of stages of frailty. Of the 29 studies included in the review, seven different aspects of mobility and PA with a multiplicity of subdivisions were examined, using 13 different sensor brands on eight different body-locations. Some studies use a combination of body-locations. This heterogeneity makes comparison and analysis difficult and thus precludes recommendations on devices. It is worth noting however that while brands of sensors reported differ, the properties are comparable. Studies will be discussed under headings referring to the various mobility and PA parameters, sensors used and body-location of sensors.

### Parameters of mobility and physical activity

#### Physical activity parameters

Time spent in non-sedentary activity is the most commonly examined parameter of mobility and PA in the literature reviewed. Subdivisions of PA patterns and PA behaviour examined include time spent in non-sedentary activity; time spent in various intensities of activity; number of postural transitions, number of bouts, length of unbroken bouts and variability in bouts of the different measurements of PA.

There was some commonality of metrics among the 12 studies in this group [11, 17, 24, 35–38, 40–43, 55] and some consensus. Razjouyan et al., [41] agree with earlier findings of Theou et al., [24] that total time spent in non-sedentary activity correlates well with a frailty index, demonstrating significant differences between levels of frailty. This is supported by Jansen et al., [43] in a study which examines the effect of frailty levels on motor capacity and mobility performance. The authors suggest that capacity does not necessarily determine performance or function but there is a strong association between the two and frailty. These findings are contradicted by Schwenk et al., [38] who suggest that percentage of time spent walking is a poor discriminator of frailty levels. These authors [38] suggest variability in walking bouts described as more static and less complex PA combined with shorter walking bouts as a more sensitive measure of frailty. Similarly, it is suggested that sedentary time is associated with frailty [36, 41] but this is refuted in another study [17].

Some studies measured intensity of PA, but as is common with many of the parameters in the studies included in this review, there is little consistency in how the metrics are defined or measured. Categories of PA intensity are consistent insofar as they are referred to as variations of low, medium or high [11, 17, 34, 36, 37, 41, 42, 44, 55] but how each category is defined differs, from measurement of acceleration counts per minute [11, 17] to metabolic equivalents (MET) [11, 36, 37, 41, 55] and magnitude of mobility e.g. lying, sitting, walking pace [42]. Counts per minute as a metric of PA intensity are not universal and there is marked disparity between the scales used [11, 17, 34, 35].

There is some agreement that moderate to vigorous activity is inversely related to frailty. Those studies that differentiate between levels of frailty agree that PA intensity discriminates NF from PF and to a lesser extent PF from F [17, 36, 37, 41, 55]. This is refuted by Jansen et al. [11] who found no significant between-group differences. The much lower counts per minute used in this study may account for this finding. Acceleration counts as measured in one study [24] are referred to as postural transitions or counts per minute (CPM) in others [34, 35, 37]. One study [40] in which postural transitions are further defined as sit to stand, stand to sit, stand to walk etc. purports the ability of the number of postural transitions to discriminate between levels of frailty while the others suggest discrimination between F and NF only [34, 35].

Within the literature included in the review, the most common correlation between frailty levels and PA demonstrated are moderate – vigorous PA (MVPA) [17, 36, 37, 41, 55], bouts of PA [38, 41, 43, 55] and total number of steps [24, 37, 41, 43, 55].

#### Temporal-spatial parameters of gait including trunk kinematics

Seven studies [24, 25, 29, 30, 40, 41, 43,] examined gait speed, velocity or time to complete a walk test as part of their research. Five included gait speed with temporal-spatial parameters including step time, regularity; stride time, length regularity; percentage of time in double support and trunk kinematics of angular velocity and trunk displacement [33, 38, 49, 50, 56]. One study examined trunk kinematics only, during the STS, Stand to Sit (St-Si) and turn transitions of 10-m Timed Up and Go (TUG) test [47, 51]. While there is consensus regarding the association between gait speed/velocity and the identification of frailty [24, 33, 38, 47, 54] there is disparity in the significance of the results. All agree on the ability of gait speed/velocity to discriminate between NF and F however the effect size varies considerably, even between studies using the same body-location [38, 54]. Variation in the methodology of gait speed measurement may be a contributory factor in the disparity, with distance over which speed was measured varying from 3 to 20 m. One study suggests that the ability to distinguish between PF and F, arguably a more important distinction, lies within the development of models including capacity and performance [43]. This study included measures of normal and fast walking speed as measures of capacity.

#### Balance

Balance is measured in different ways throughout the literature varying in the nature of the assessment, the conditions under which the assessment took place and duration of each task. Those that assessed balance during a period of quiet standing did so over different time periods ranging from 10 – 40-s [38, 45, 49, 57]. Conditions varied between participants standing with feet together, feet semi-tandem, eyes open and/or eyes closed while another measured balance during a 30-s chair-stand exercise [46]. Balance was evaluated by examining displacement of trunk [38, 45, 46, 49], hip and ankle [38, 57] in anteroposterior and medial–lateral directions and during different phases of the task [46].

Studies that investigated the effect of balance parameters on the identification of frailty agree on a greater anteroposterior sway in frail groups under conditions of feet together, eyes closed but no between-group significance [38, 45, 57]. Millor et al., [46] concur to some extent in their assessment of lateral sway. However synthesis of data is difficult because of the study characteristics. These studies varied greatly in their methodology and analysis. One study [45] proposes analysis of the orientation and acceleration signal-intensity as a novel and perhaps more appropriate approach to discriminating between frailty levels than sway or power variables of balance tests. Results of this study indicate that the higher frequencies of orientation and acceleration signals obtained through wavelet decomposition analysis in healthy populations are distinguished from the lower frequencies typical of a frail population.

One study that examined a broad range of variables suggests that the predictive validity of balance parameters is inferior to those of gait and PA parameters [38]. Subsequently it has been suggested that kinematics of STS have greater sensitivity, specificity, accuracy and precision values than those of gait parameters, specifically velocity [58]. This is supported by one study which, using a model combining data from balance, PA and chair kinematics, yields a higher accuracy percentage in identifying frailty than each of the individual tests [49].

#### Upper limb kinematics

Three studies [39, 52, 53] examined kinematics of the upper limb, specifically the elbow, in the development of a frailty assessment tool that does not rely on gait. All agree on the ability of the variables derived from an elbow flexion/extension task to distinguish between levels of frailty.

### Sensors and body-location

With the exception of two studies [24, 37] in which a uni-axial accelerometer was used, all studies report the use of either a tri-axial accelerometer, gyroscope or a combination of both, with the inclusion of a tri-axial magnetometer reported in eight studies [33, 45–48, 54, 56, 58]. The uni-axial accelerometer was positioned at the waist and used to record steps in conjunction with acceleration counts [24] and total number of steps with PA intensity [37]. The most common body-location for the tri-axial sensors was the lumbar spine [38, 43, 45, 46, 49, 56–58], but in other studies these sensors were positioned at the chest [24, 40, 41, 47–49, 51], shins [33, 38, 43, 50, 54, 57, 60], wrist [35, 39, 42, 52, 53], waist [11, 55], hip [17, 36] thigh [33, 38] and foot [33].

There was some commonality with the body-locations used and metrics obtained, for example all balance parameters were obtained using a tri-axial gyroscope positioned at the LSp [38, 45, 46, 57, 60]. However in some studies a sensor positioned at the LSp was used to examine temporal-spatial parameters of gait [56, 58]. One study used a combination of LSp and shin to measure balance parameters, presumably because the study examined open-loop and closed-loop postural control strategy [57].

Body-location of sensors measuring PA included chest [38, 40, 41, 43, 51, 60], wrist [35, 42], hip [17, 36] and waist [24, 55]. One study in this group [38] used a combination of body-locations but reports that data for PA was retrieved from only the sensor located at the chest.

Correlation between accelerometer counts and step counts in one study [24] was less in the higher FI cohort, which is surprising considering both were obtained from the same device. This perhaps suggests less sensitivity in accelerometers in detecting lower intensity of movement. This supports the idea mooted that activity below a cut-off point considered in some research as non-wear time may in fact reflect low intensity activity [61]. The same study [24] found that minute-by-minute accelerometer-derived step-count and acceleration-counts correlated positively with HR values. This is interesting considering as referred to previously, heart rate monitors capture indications of physical activities that do not require trunk displacement and can be used to indicate energy expenditure and physical activity behaviours e.g. sedentary time.

## Limitations

While every effort has been made to ensure a thorough search of the relevant databases it is possible that some literature was missed. An updated search performed prior to journal submission reduces the risk of any over-sight. The inclusion of English-only publications may have resulted in omission of some relevant studies. Applying the age profile criteria of > 60 years in the inclusion may be perceived as a limitation but this was done to optimise the literature included and is in accordance with the World Health Organization and the United Nations who have adopted > 60 years in reference to older adults as opposed to the arbitrary 65 years commonly adopted [62]. Due to the heterogeneity of metrics, the variation in body-location of sensor placement and the difference in methods of analysis among the studies included in the review, meta-analysis was not possible. This however does not invalidate the findings. Many studies involved small numbers of participants and some combined frail and pre-frail cohorts for statistical analysis. This reduces the potential to discriminate between levels of frailty which is considered an important objective.

## Conclusions

Despite its limitations, this review, the first to comprehensively synthesise data from the last decade of research in this field, makes a valuable contribution to identifying how wearable sensors have been utilised to assess frailty in older adults, the body-locations of sensor-placement used and the parameters of PA and mobility that best assist in the discrimination of frailty levels. The review highlights the heterogeneity of parameters examined in relation to frailty identification and the body-locations used. Measurements of PA have proved to be the most frequently used parameter when all variations of number of postural transitions, number of steps, percentage of time in PA and intensity of PA are considered. Only one study failed to demonstrate an association between PA and levels of frailty. Gait-speed was found to be the next most prevalent parameter examined, with all studies included in the review demonstrating a correlation between walking speed and levels of frailty. A higher sensitivity compared with other mobility parameters is noted.

Considering the facts that up to ninety-five percent of older adults are community-dwelling, that not all older adults develop frailty and that research suggests older adults can transition between levels of frailty, this review highlights the need for further research to identify a feasible, user-friendly device and body-location that can be used to independently identify and objectively measure signs of pre-frailty in community-dwelling older adults. This could facilitate early identification and targeted intervention to reduce the burden of frailty in an ageing population. Future reviews could focus on important open research questions related to wearable technology and older adults including acceptance, feasibility and facilitation of ageing in place.

## Data Availability

Not applicable.

## Appendix 1. Medline (Ebsco) Search strategy / terms

Search Alert: "AB ( elderly OR aged OR older OR elder OR geriatric OR elderly people OR old people OR senior) AND AB ( frailty OR frail OR “frailty syndrome”) AND AB ( wearable technology OR wearable devices OR body-worn sensor OR inertial sensor OR inertial measurement unit OR IMU OR accelerometer OR accelerometry OR actigraphy OR pedometer OR activity monitor OR daily steps OR GPS OR global positioning system OR activity tracker OR fitness trackers OR physical activity tracking OR physical fitness tracker OR biosensing OR biosensor) AND AB ( physical activity OR physical function OR mobility OR gait OR walking OR ambulation OR function OR locomotion OR mobility OR speed OR postural transition OR sit to stand OR chair stand) AND AB ( validity OR validation OR validation study OR reliability OR reliability study OR accuracy OR comparison OR comparison study) Date of Publication: 20,100,101–20,201,231 AND Apply equivalent subjects on 2020–03-31 06:13 AM".

## Appendix 2. Excluded studies

**Table:**

Author and year	Reason for exclusion
Mueller [67]	Proof of concept study. Doesn’t use parameters to identify frailty
Keppler [68]	Not frailty
Chigateri [69]	Comparing algorithm with video
Soaz [70]	Validation of step-detection algorithm
Fontecha [71]	Development of app
Da Silva [72]	Used non-wearable sensors
Chkeir [73]	Used non-wearable sensors
Thiede [66]	Population studied aged < 60 year
Zhong [74]	Population studied aged < 60 year
Rahemi [75]	Population studied aged < 60 year
Martinez-Ramirez [76]	Population studied included people with cognitive impairment

## Appendix 3. AXIS TOOL

### AXIS Critical Appraisal Tool Yes [1] / No [0] / Don’t Know [0]

#### Introduction

1Were the aims/objectives of the study clear?

#### Methods

2 Was the study design appropriate for the stated aim(s)?

3 Was the sample size justified?

4 Was the target/reference population clearly defined? (Is it clear who the research was about?).

5 Was the sample frame taken from an appropriate population base so that it closely represented the target/reference population under investigation?

6 Was the selection process likely to select subjects/participants that were representative of the target/reference population under investigation?

7 Were measures undertaken to address and categorise non-responders?

8 Were the frailty assessment tool and outcome variables measured appropriate to the aims of the study?

9 Were the frailty assessment tool and outcome variables measured correctly using instruments/ measurements that had been trialled, piloted or published previously?

10 Is it clear what was used to determined statistical significance and/or precision estimates? (e.g., p values, CIs).

11 Were the methods (including statistical methods) sufficiently described to enable them to be repeated?

#### Results

12 Were the basic data adequately described?

13 *Does the response rate raise concerns about non-response bias?

14 If appropriate, was information about non-responders described?

15 Were the results internally consistent?

16 Were the results for the analyses described in the methods, presented?

#### Discussion

17 Were the authors’ discussions and conclusions justified by the results?

18 Were the limitations of the study discussed? Other.

19 *Were there any funding sources or conflicts of interest that may affect the authors’ interpretation of the results?

20 Was ethical approval or consent of participants attained?

*Negative answer results in ‘Y’ Yes = 0; No = 1.

## Abbreviations

AXIS: Appraisal Tool for Cross-sectional Studies
CPM: Counts per minute
F: Frailty
FI: Frailty Index
FFP: Fried’s Frailty Phenotype
ISAR-HP: Identification Seniors At Risk-Hospitalized Patients’ questionnaire
LSp: Lumbar Spine
MVPA: Moderate – Vigorous Physical Activity
NF: Non-Frail
PICO: Population, Intervention, Comparator and Outcome
PF: Pre-Frail
PRISMA: Preferred Reporting Items for Systematic Reviews and Meta-Analyses
PROSPERO: Prospective Register Of Systematic Reviews
STS: Sit To Stand
St-Si: Stand to Sit
TUG: Timed Up and Go

## References

[1] UN Department of Economics and Social Affairs (2015). World population prospects—population division—United Nations. Int J Logist Manag.

[2] Fried LP, Tangen CM, Walston J, Newman AB, Hirsch C, Gottdiener J (2001). Frailty in older adults: evidence for a phenotype. Journals Gerontol Ser A Biol Sci Med Sci.

[3] Buta BJ, Walston JD, Godino JG, Park M, Kalyani RR, Xue QL (2016). Frailty assessment instruments: systematic characterization of the uses and contexts of highly-cited instruments. Ageing Res Rev.

[4] De Vries NM, Staal JB, Van Ravensberg CD, Hobbelen JSM, Rikkert MGMO, Nijhuis-Van Der Sanden MWG. Outcome instruments to measure frailty: A systematic review. Ageing Res Rev. 2010;10:104–14. 10.1016/j.arr.2010.09.001 20850567

[5] O’Halloran A, O’Shea M (2018). Wellbeing and health in Ireland’s over 50s 2009–2016 Chapter 7: frailty. TILDA..

[6] Ofori-Asenso R, Chin KL, Mazidi M, Zomer E, Ilomaki J, Zullo AR (2019). Global incidence of frailty and prefrailty among community-dwelling older adults: a systematic review and meta-analysis. JAMA Netw Open.

[7] Kojima G, Taniguchi Y, Iliffe S, Jivraj S, Walters K (2019). Transitions between frailty states among community-dwelling older people: a systematic review and meta-analysis. Ageing Res Rev.

[8] O ’caoimh R, Galluzzo L, Van Der Heyden J, Carriazo AM, Samaniego LL, Koula M, et al. Title: frailty at population level: a systematic review [Internet]. 2017. http://advantageja.eu/images/WP5-Frailty-at-Population-Level-a-Systematic-Review-.pdf. Accessed 6 Jan 2020.

[9] Zhang Q, Guo H, Gu H, Zhao X (2018). Gender-associated factors for frailty and their impact on hospitalization and mortality among community- dwelling older adults: a cross-sectional population-based study. PeerJ.

[10] Song J, Lindquist LA, Chang RW, Semanik PA, Ehrlich-Jones LS, Lee J (2015). Sedentary behavior as a risk factor for physical frailty independent of moderate activity: results from the osteoarthritis initiative. Am J Public Health.

[11] Jansen FM, Prins RG, Etman A, van der Ploeg HP, de Vries SI, van Lenthe FJ (2015). Physical activity in non-frail and frail older adults. PLoS ONE.

[12] Blodgett J, Theou O, Kirkland S, Andreou P, Rockwood K (2015). The association between sedentary behaviour, moderate-vigorousphysical activity and frailty in NHANES cohorts. Maturitas.

[13] Lewis EG, Coles S, Howorth K, Kissima J, Gray W, Urasa S (2018). The prevalence and characteristics of frailty by frailty phenotype in rural Tanzania. BMC Geriatr.

[14] Warburton DER, Bredin SSD (2016). Reflections on physical activity and health: what should we recommend?. Can J Cardiol.

[15] World Health Organization (2018). Global action plan on physical activity 2018–2030: more active people for a healthier world.

[16] World Health Organization (2020). WHO Guidelines on physical activity, sedentary behaviour.

[17] Castaneda-Gameros D, Redwood S, Thompson JL (2018). Physical activity, sedentary time, and frailty in older migrant women from ethnically diverse backgrounds: a mixed-methods study. J Aging Phys Act.

[18] Hurtig-Wennlf A, Hagstrmer M, Olsson LA (2010). The International Physical Activity Questionnaire modified for the elderly: aspects of validity and feasibility. Public Health Nutr.

[19] Sylvia LG, Bernstein EE, Hubbard JL, Keating L, Anderson EJ (2014). Practical guide to measuring physical activity. J Acad Nutr Diet.

[20] Doherty A, Jackson D, Hammerla N, Plötz T, Olivier P, Granat MH (2017). Large scale population assessment of physical activity using wrist worn accelerometers: the UK Biobank Study. PLoS ONE.

[21] Straiton N, Alharbi M, Bauman A, Neubeck L, Gullick J, Bhindi R (2018). The validity and reliability of consumer-grade activity trackers in older, community-dwelling adults: a systematic review. Maturitas.

[22] Zampogna A, Mileti I, Palermo E, Celletti C, Paoloni M, Manoni A (2020). Fifteen years of wireless sensors for balance assessment in neurological disorders. Sensors (Switzerland).

[23] O’Neill B, McDonough SM, Wilson JJ, Bradbury I, Hayes K, Kirk A (2017). Comparing accelerometer, pedometer and a questionnaire for measuring physical activity in bronchiectasis: a validity and feasibility study. Respir Res.

[24] Theou O, Jakobi JM, Vandervoort AA, Jones GR (2012). A comparison of physical activity (PA) assessment tools across levels of frailty. Arch Gerontol Geriatr.

[25] CSO. Census of population 2016 [Internet]. 2019. https://www.cso.ie/en/releasesandpublications/ep/p-cp9hdc/p8hdc/p9tod/. Accessed 6 Jan 2020.

[26] Schwenk M, Howe C, Saleh A, Mohler J, Grewal G, Armstrong D (2013). Frailty and technology: A systematic review of gait analysis in those with frailty. Gerontology.

[27] Pang I, Okubo Y, Sturnieks D, Lord SR, Brodie MA (2019). Detection of near falls using wearable devices: a systematic review. J Geriatr Phys Ther.

[28] Patel S, Park H, Bonato P, Chan L, Rodgers M (2012). A review of wearable sensors and systems with application in rehabilitation. J NeuroEngineering Rehabil..

[29] McCullagh R, Brady NM, Dillon C, Frances Horgan N, Timmons S (2016). A review of the accuracy and utility of motion sensors to measure physical activity of frail, older hospitalized patients. J Aging Phys Act.

[30] Moher D, Liberati A, Tetzlaff J, Altman DG, Altman D, Antes G (2009). Preferred reporting items for systematic reviews and meta-analyses: the PRISMA statement. PLoS Med.

[31] Binotto MA, Lenardt MH, Del Carmen R-M (2018). Physical frailty and gait speed in community elderly: a systematic review. Rev Esc Enferm USP.

[32] Downes MJ, Brennan ML, Williams HC, Dean RS (2016). Appraisal tool for Cross-Sectional Studies (AXIS). BMJ Open.

[33] Apsega A, Petrauskas L, Alekna V, Daunoraviciene K, Sevcenko V, Mastaviciute A (2020). Wearable sensors technology as a tool for discriminating frailty levels during instrumented gait analysis. Appl Sci.

[34] Ziller C, Braun T, Thiel C (2020). Frailty phenotype prevalence in community-dwelling older adults according to physical activity assessment method. Clin Interv Aging.

[35] Huisingh-Scheetz M, Wroblewski K, Kocherginsky M, Huang E, Dale W, Waite L (2018). The relationship between physical activity and frailty among U.S. older adults based on hourly accelerometry data. J Gerontol Ser A Biol Sci Med Sci.

[36] Kikuchi H, Inoue S, Amagasa S, Fukushima N, Machida M, Murayama H (2020). Associations of older adults’ physical activity and bout-specific sedentary time with frailty status: Compositional analyses from the NEIGE study. Exp Gerontol.

[37] Yuki A, Otsuka R, Tange C, Nishita Y, Tomida M, Ando F (2019). Daily physical activity predicts frailty development among community-dwelling older japanese adults. J Am Med Dir Assoc.

[38] Schwenk M, Mohler J, Wendel C, D’Huyvetter K, Fain M, Taylor-Piliae R (2015). Wearable sensor-based in-home assessment of gait, balance, and physical activity for discrimination of frailty status: baseline results of the Arizona frailty cohort study. Gerontology.

[39] Toosizadeh N, Mohler J, Najafi B (2015). Assessing upper extremity motion: An innovative method to identify frailty. J Am Geriatr Soc.

[40] Parvaneh S, Mohler J, Toosizadeh N, Grewal GS, Najafi B (2017). Postural transitions during activities of daily living could identify frailty status: application of wearable technology to identify frailty during unsupervised condition. Gerontology.

[41] Razjouyan J, Naik AD, Horstman MJ, Kunik ME, Amirmazaheri M, Zhou H (2018). Wearable sensors and the assessment of frailty among vulnerable older adults: an observational cohort study. Sensors.

[42] Mulasso A, Brustio PR, Rainoldi A, Zia G, Feletti L, N’Dja A (2019). A comparison between an ICT tool and a traditional physical measure for frailty evaluation in older adults. BMC Geriatr.

[43] Jansen CP, Toosizadeh N, Mohler MJ, Najafi B, Wendel C, Schwenk M (2019). The association between motor capacity and mobility performance: frailty as a moderator. Eur Rev Aging Phys Act.

[44] Chen S, Honda T, Chen T, Narazaki K, Haeuchi Y, Supartini A (2015). Screening for frailty phenotype with objectively-measured physical activity in a west Japanese suburban community: evidence from the Sasaguri Genkimon Study. BMC Geriatr.

[45] Martınez-Ramırez A, Lecumberri P, Gomez M, Rodriguez-Manas L, Garcıa FJ, Izquierdo M (2011). Frailty assessment based on wavelet analysis during quiet standing balance test. J Biomech.

[46] Millor N, Lecumberri P, Gómez M, Martínez-Ramírez A, Izquierdo M (2013). An evaluation of the 30-s chair stand test in older adults: frailty detection based on kinematic parameters from a single inertial unit. J Neuroeng Rehabil.

[47] Galán-mercant A, Cuesta-vargas AI (2013). Differences in trunk kinematic between frail and nonfrail elderly persons during turn transition based on a smartphone inertial sensor. Biomed Res Int.

[48] Lepetit K, Mansour KB, Letocart A, Boudaoud S, Kinugawa K, Grosset J-F (2019). Optimized scoring tool to quantify the functional performance during the sit-to-stand transition with a magneto-inertial measurement unit. Clin Biomech.

[49] Greene BR, Doheny EP, Kenny RA, Caulfield B (2014). Classification of frailty and falls history using a combination of sensor-based mobility assessments. Physiol Meas.

[50] Greene BR, Doheny EP, Kenny RA, O’Halloran A (2014). Frailty status can be accurately assessed using inertial sensors and the TUG test. Age Ageing.

[51] Galán-Mercant A, Cuesta-Vargas AI (2013). Differences in trunk accelerometry between frail and nonfrail elderly persons in sit-to-stand and stand-to-sit transitions based on a mobile inertial sensor. J Med Internet Res.

[52] Lee H, Joseph B, Enriquez A, Najafi B (2018). Toward using a smartwatch to monitor frailty in a hospital setting: Using a single wrist-wearable sensor to assess frailty in Bedbound inpatients. Gerontology.

[53] Toosizadeh N, Joseph B, Heusser MR, Orouji Jokar T, Mohler J, Phelan HA (2016). Assessing upper-extremity motion: an innovative, objective method to identify frailty in older bed-bound trauma patients. J Am Coll Surg.

[54] Zhou H, Razjouyan J, Halder D, Naik AD, Kunik ME, Najafi B (2019). Instrumented trail-making task: application of wearable sensor to determine physical frailty phenotypes. Gerontology.

[55] Chen S, Chen T, Kishimoto H, Yatsugi H, Kumagai S (2020). Associations of objectively measured patterns of sedentary behavior and physical activity with frailty status screened by the frail scale in Japanese community-dwelling older adults. J Sport Sci Med.

[56] Martínez-Ramírez A, Martinikorena I, Gómez M, Lecumberri P, Millor N, Rodríguez-Mañas L (2015). Frailty assessment based on trunk kinematic parameters during walking. J Neuroeng Rehabil.

[57] Toosizadeh N, Mohler J, Wendel C, Najafi B (2015). Influences of frailty syndrome on open-loop and closed-loop postural control strategy. Gerontology.

[58] Millor N, Lecumberri P, Gomez M, Martinez A, Martinikorena J, Rodriguez-Manas L (2017). Gait velocity and chair sit-stand-sit performance improves current frailty-status identification. IEEE Trans Neural Syst Rehabil Eng.

[59] Downes MJ, Brennan ML, Williams HC, Dean RS (2016). Development of a critical appraisal tool to assess the quality of cross-sectional studies (AXIS). BMJ Open.

[60] Greene BR, Doheny EP, Walsh C, Cunningham C, Crosby L, Kenny RA (2012). Evaluation of falls risk in community-dwelling older adults using body-worn sensors. Gerontology.

[61] Gorman E, Hanson HM, Yang PH, Khan KM, Liu-Ambrose T, Ashe MC (2014). Accelerometry analysis of physical activity and sedentary behavior in older adults: a systematic review and data analysis. Eur Rev Aging Phys Act.

[62] World Health Organization (2015). World report on ageing and health 2015.

[63] Greene BR, Odonovan A, Romero-Ortuno R, Cogan L, Scanaill CN, Kenny RA (2010). Quantitative falls risk assessment using the timed up and go test. IEEE Trans Biomed Eng.

[64] Doheny EP, Greene BR, Foran T, Cunningham C, Fan CW, Kenny RA (2012). Diurnal variations in the outcomes of instrumented gait and quiet standing balance assessments and their association with falls history. Physiol Meas.

[65] Doheny EP, Walsh C, Foran T, Greene BR, Fan CW, Cunningham C (2013). Falls classification using tri-axial accelerometers during the five-times-sit-to-stand test. Gait Posture.

[66] Thiede R, Toosizadeh N, Mills JL, Zaky M, Mohler J, Najafi B (2016). Gait and balance assessments as early indicators of frailty in patients with known peripheral artery disease. Clin Biomech.

[67] Mueller A, Hoefling HA, Muaremi A, Praestgaard J, Walsh LC, Bunte O (2019). Continuous digital monitoring of walking speed in frail elderly patients: noninterventional validation study and longitudinal clinical trial. JMIR mHealth uHealth.

[68] Keppler AM, Nuritidinow T, Mueller A, Hoefling H, Schieker M, Clay I (2019). Validity of accelerometry in step detection and gait speed measurement in orthogeriatric patients. PLoS ONE.

[69] Chigateri NG, Kerse N, Wheeler L, MacDonald B, Klenk J (2018). Validation of an accelerometer for measurement of activity in frail older people. Gait Posture.

[70] Soaz C, Diepold K (2016). Step detection and parameterization for gait assessment using a single waist-worn accelerometer. IEEE Trans Biomed Eng.

[71] Fontecha J, Hervás R, Bravo J, Navarro FJ (2013). A mobile and ubiquitous approach for supporting frailty assessment in elderly people. J Med Internet Res.

[72] Da Silva VD, Tribess S, Meneguci J, Sasaki JE, Garcia-Meneguci CA, Carneiro JAO (2019). Association between frailty and the combination of physical activity level and sedentary behavior in older adults. BMC Public Health.

[73] Chkeir A, Novella JL, Dramé M, Bera D, Collart M, Duchêne J (2019). In-home physical frailty monitoring: Relevance with respect to clinical tests. BMC Geriatr.

[74] Zhong R, Rau P-LP, Yan X (2018). Application of smart bracelet to monitor frailty-related gait parameters of older Chinese adults: a preliminary study. Geriatr Gerontol Int.

[75] Rahemi H, Nguyen H, Lee H, Najafi B, Lee H (2018). Toward smart footwear to track frailty phenotypes-using propulsion performance to determine frailty. Sensors.

[76] Martínez-Ramírez A, Martinikorena I, Lecumberri P, Gómez M, Millor N, Casas-Herrero A (2016). Dual task gait performance in frail individuals with and without mild cognitive impairment. Dement Geriatr Cogn Disord.

